# Cooperativity Between CD8+ T Cells, Non-Neutralizing Antibodies, and Alveolar Macrophages Is Important for Heterosubtypic Influenza Virus Immunity

**DOI:** 10.1371/journal.ppat.1003207

**Published:** 2013-03-14

**Authors:** Brian J. Laidlaw, Vilma Decman, Mohammed-Alkhatim A. Ali, Michael C. Abt, Amaya I. Wolf, Laurel A. Monticelli, Krystyna Mozdzanowska, Jill M. Angelosanto, David Artis, Jan Erikson, E. John Wherry

**Affiliations:** 1 Department of Microbiology and Institute for Immunology, University of Pennsylvania School of Medicine, Philadelphia, Pennsylvania, United States of America; 2 The Wistar Institute, Philadelphia, Pennsylvania, United States of America; National Institutes of Health, United States of America

## Abstract

Seasonal epidemics of influenza virus result in ∼36,000 deaths annually in the United States. Current vaccines against influenza virus elicit an antibody response specific for the envelope glycoproteins. However, high mutation rates result in the emergence of new viral serotypes, which elude neutralization by preexisting antibodies. T lymphocytes have been reported to be capable of mediating heterosubtypic protection through recognition of internal, more conserved, influenza virus proteins. Here, we demonstrate using a recombinant influenza virus expressing the LCMV GP33-41 epitope that influenza virus-specific CD8+ T cells and virus-specific non-neutralizing antibodies each are relatively ineffective at conferring heterosubtypic protective immunity alone. However, when combined virus-specific CD8 T cells and non-neutralizing antibodies cooperatively elicit robust protective immunity. This synergistic improvement in protective immunity is dependent, at least in part, on alveolar macrophages and/or other lung phagocytes. Overall, our studies suggest that an influenza vaccine capable of eliciting both CD8+ T cells and antibodies specific for highly conserved influenza proteins may be able to provide heterosubtypic protection in humans, and act as the basis for a potential “universal” vaccine.

## Author Summary

Influenza virus continues to pose a significant risk to global health and is responsible for thousands of deaths each year in the United States. This threat is largely due to the ability of the influenza virus to undergo rapid changes, allowing it to escape from immune responses elicited by previous infections or vaccinations. Certain internal determinants of the influenza virus are largely conserved across different viral strains and represent attractive targets for potential “universal” influenza vaccines. Here, we demonstrated that cross-subtype protection against the influenza virus could be obtained through simultaneous priming of multiple arms of the immune response against conserved elements of the influenza virus. These results suggest a novel strategy that could potentially form a primary component of a universal influenza vaccine capable of providing long-lasting protection.

## Introduction

Influenza virus remains a significant threat to global health, and results in 200,000 hospitalizations and 3,000–49,000 deaths each year in the United States [1]–[3]. The ability of influenza virus to rapidly mutate and/or undergo reassortment, allows the virus to evade protective immunity obtained from previous infections or vaccinations [4]. Annual influenza vaccines induce an antibody response specific for the highly variable surface glycoproteins of influenza: neuraminidase (NA) and hemagglutinin (HA). These seasonal vaccines typically take months to produce and rely on the accurate prediction of the influenza serotypes that will be circulating in the next flu season [5]. Thus, if the prediction is not accurate or a pandemic strain emerges, current vaccines offer little protection. Much research has therefore, focused on the development of a “universal” vaccine that will target the conserved, internal regions of the influenza virus, and confer protection against multiple influenza virus serotypes.

Significant research in the influenza field has focused on the design of vaccines capable of eliciting influenza virus-specific CD8+ T lymphocytes [6]–[8]. Since, CD8+ T cells are able to recognize internal, conserved regions of the influenza virus, these cells may be able provide cross-subtype, or heterosubtypic, protection against the influenza virus [9]–[13]. A large body of work supports the potential viability of a CD8+ T-cell based vaccine [14]–[17]. In mice, vaccination with internal proteins such as influenza nucleoprotein (NP), leads to higher frequencies of NP-specific CD8+ T cells prior to infection, and lower viral titers after challenge with H1N1 and H3N2 strains of influenza [18]–[22]. Furthermore, influenza virus-specific memory T cells are detected in the peripheral blood of healthy adolescents and adults, and there is some evidence for heterosubtypic immunity in humans that has been proposed to be due to T cells [23]–[25].

However, several groups have reported that the number of influenza virus-specific memory CD8+ T cells in the lung airways of mice declines over time corresponding with a loss of heterosubtypic protection [26]–[29]. While there is some conflicting data over whether heterosubtypic protection wanes, if true this gradual loss of the virus-specific CD8+ T cell population represents a serious concern in the generation of CD8+ T cell based vaccines [30]. Interestingly, recent work suggests that non-neutralizing antibodies targeting the internal proteins of influenza, specifically NP, can provide some protection against the influenza virus infection through a mechanism involving Fc receptors [31]–[34]. Unlike neutralizing antibodies, which are able to prevent viral entry or exit, non-neutralizing antibodies typically target antigens that reside inside virions and/or infected cells. Despite recent progress, it is still not clear what role T cells play in non neutralizing antibody-mediated heterosubtypic protection elicited in an immune competent host. Additionally the mechanisms by which non-neutralizing antibodies can provide protection against the influenza virus remain elusive.

Several groups have also noted the potential role of CD4+ T cells in providing protection against influenza virus [6], [35], [36]. These reports indicated that CD4+ T cells can form a lung-resident population following influenza virus infection where they can serve a protective role in mediating enhanced viral clearance and survival following lethal challenge through a variety of mechanisms including IFNγ secretion [35], [37]. Recent studies using human volunteers infected with influenza virus also point to a key role for pre-existing CD4 T cell responses in limiting the severity of influenza virus infection and disease [38]. Intriguingly, influenza-specific memory CD4+ T cells have also been reported to synergize with naïve B cells and CD8+ T cells to provide protection against influenza viral infection [36]. Whether virus-specific CD8 T cells also exhibit such cooperativity in protective immunity is unclear.

In this study, we demonstrate that, in most settings, influenza virus-specific CD8+ T cells alone are insufficient to provide optimal protection against influenza virus. However, when virus-specific non-neutralizing antibodies are present together with virus-specific CD8+ T cells, complete protection is achieved against a lethal influenza virus challenge. Moreover, this cooperative protection is dependent, at least in part, on the presence of alveolar macrophages (AM) or other respiratory tract phagocytes, suggesting that non-neutralizing antibodies are able to eliminate influenza virus-infected cells through antibody-dependent cell-mediated cytotoxicity (ADCC) and/or phagocytosis. We demonstrate a novel mechanism by which antibodies and CD8+ T cells targeted against the conserved regions of the influenza virus act in concert to provide heterosubtypic protection. Our results complement recent work on the synergy between memory CD4+ T cells and naïve B and CD8 T cells [36] and suggest that elicitating multiple arms of the adaptive immune response may represent a potent mechanism by which heterosubtypic protection against the influenza virus can be achieved.

## Results

### Magnitude of influenza virus-specific CD8+ T cell response is not indicative of protection

It has been reported that CD8+ T cell activity correlates with reduced influenza virus shedding following rechallenge [13]. Since, CD8+ T cell epitopes are often located in the internal, conserved regions of the influenza virus, the generation of influenza virus-specific CD8+ T cells may provide protective immunity against heterosubtypic influenza strains. Thus, we tested whether influenza virus-specific CD8+ T cells could mediate protective immunity using a recombinant viral approach to identify and track responses. We used influenza viruses in which the GP33-41 epitope from lymphocytic choriomeningitis virus (LCMV), was inserted into the NA stalk region of the H3N2 influenza X31 (X31-GP33) and H1N1 influenza PR8 (PR8-GP33) viruses [39], [40]. Influenza viruses expressing the GP33 epitope have been shown to induce a robust GP33 response in mice [39], [41]. Mice were primed with either LCMV Armstrong or X31-GP33 intranasally (i.n.) and rechallenged, along with a control group of naïve mice, with PR8-GP33 30 days later. The antibodies generated against the surface glycoproteins of the H3N2 X31-GP33 virus do not neutralize the H1N1 PR8-GP33 challenge virus [42]–[44]. The GP33-specific CD8+ T cell population elicited from primary viral challenge, however, should be capable of responding to the secondary infection, allowing the role of CD8+ T cells in protection against the influenza virus to be investigated.

**Figure 1:**
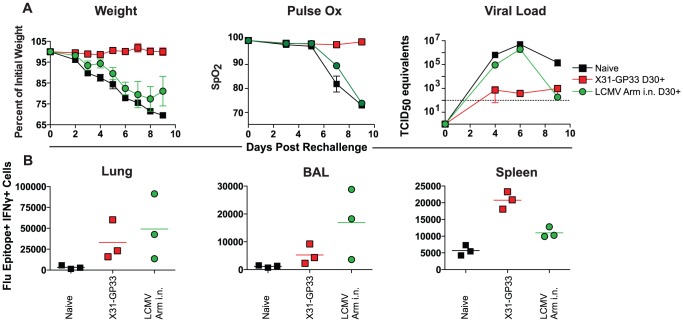
Protection against influenza virus rechallenge appears to be independent of magnitude of virus-specific CD8+ T cell response. A) C57BL/6 mice were primed with either X31-GP33 i.n. or LCMV Armstrong i.n. These mice, as well as a naïve control group, were rechallenged with 3 LD_50_ influenza PR8-GP33 virus on day 30+ following primary infection. Weight loss after rechallenge and lung function (as measured by pulse oximetry) were assessed. Mice were sacrificed on day 0, 4, 6, and 9 post rechallenge and viral titers were determined in the lungs. Data are representative of 9 mice per group with three mice sacrificed at each time point for viral load determination. B) The lung, spleen and BAL were collected on day 6 to assess the influenza virus-specific CD8+ T cell response. Following isolation, cells were stimulated using peptide pools from the influenza proteins HA, NA, NS1/2, PA, PB, NP, as well as the LCMV GP33 peptide. Intracellular staining (ICS) for IFNγin the lung, BAL, and spleen assessed the total virus-specific CD8+ T cell response. Mice were anaesthetized using avertin. Results are representative of two independent experiments.

The level of protection conferred upon secondary challenge was determined using three assays. Morbidity was assessed by weight loss. Pulse oximetry was also used to evaluate lung function. Finally, real time quantitative (qRT-PCR) was used to detect viral RNA and determine viral load at several time points following infection. X31-GP33 primed mice were completely protected from influenza rechallenge by all 3 measures (Fig. 1A). These mice experienced almost no impairment of lung function or loss in weight, and had low viral load at all time points measured. In contrast, the LCMV Armstrong immunized mice, despite generating a robust GP33-specific CD8+ T cell response, exhibited little if any protection from the PR8-GP33 rechallenge. Apart from a slight delay in compromised lung function, the LCMV Armstrong immune mice were indistinguishable from the naïve group and experienced a 25% decline in weight and lung function by day 9 post rechallenge. To determine if the difference in protection between the X31-GP33 and LCMV Armstrong immune groups was due to the X31-GP33 immune mice having a larger influenza virus-specific CD8+ T cell response, we quantified the total immune response in these mice 6 days after rechallenge in the lung, bronchoalveolar lavage (BAL), and spleen. Responses were analyzed using intracellular staining to evaluate the number of cells in these mice able to produce interferon gamma (IFNγ) in response to stimulation with overlapping peptide pools for the influenza virus proteins HA, NA, non-structural protein 1 (NS1), NS2, polymerase acidic (PA), polymerase basic (PB), NP, as well as the LCMV GP33 peptide. We found that the LCMV Armstrong immune mice had a similar or slightly larger antiviral CD8+ T cell response directed against the recombinant influenza virus following rechallenge in both the lung and BAL despite lack of protection (Fig. 1B). Similar results were obtained using mice immunized intraperitoneally (i.p.) with LCMV Armstrong (Fig. S1A, S1B, S1C), though LCMV i.n. immunized mice had slightly enhanced viral control compared to the LCMV i.p. primed mice (Fig. S2). Overall, while the route of priming may have some impact, these results indicate that the magnitude of the virus-specific CD8+ T cell response alone might not be a major determinate of protection against influenza viral challenge and suggest that other factors could be responsible for protection against influenza virus in X31-GP33 immune mice.

### Influenza virus-specific CD8+ T cells alone are insufficient for optimal protection

We first sought to evaluate whether the agent used to prime the mice could have an impact on protection in our system. The specific priming agent used has been reported to confer differences in protection against influenza virus in several vaccine studies [45], [46]. Thus, mice were primed with LCMV Armstrong, Listeria-GP33 (LM-GP33), or Vaccinia-GP33. Each group of mice had a similar GP33-specific CD8+ T cell population despite being immunized with different bacterial or viral agents. We found that regardless of the priming agent used, all groups experienced severe weight loss, decline in lung function, and high viral load following rechallenge with PR8-GP33 (data not shown). Thus, in this setting, the priming agent did not have an obvious direct correlation with whether or not protection was achieved.

**Figure 2:**
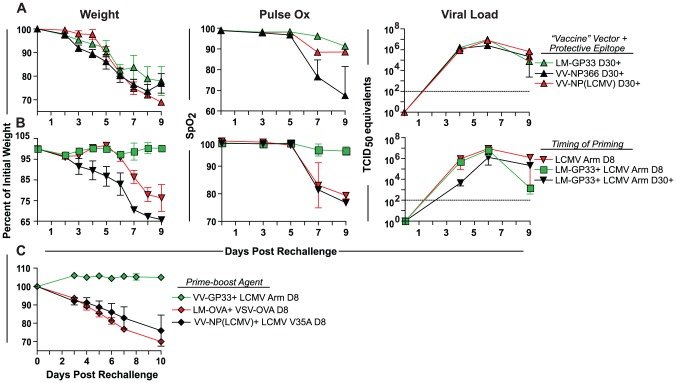
*In vivo* primed CD8+ T cells alone are insufficient for optimal influenza rechallenge protection. To determine if CD8+ T cells alone can mediate influenza protection, mice were primed using different strategies, and rechallenged with 3 LD_50_ PR8-GP33. Body weight, lung function, and viral load were determined following rechallenge. A) Mice were primed with the indicated viruses expressing GP33 or NP366. As a control, a virus expressing a non-influenza virus determinant was used (LCMV NP). Data are representative of 9 mice per group with three mice sacrificed at each time point for viral load determination. B) Mice were immunized using the indicated prime-boost strategy and rechallenged at either day 8 or day 30 following the boost. Additionally, mice were rechallenged at effector time point (d8) following primary infection with LCMV Arm i.n. Data are representative of 9 mice per group with three mice sacrificed at each time point for viral load determination. C) A prime-boost approach that elicited a CD8+ T cell response to either GP33 or a non-influenza epitope was also used. For the latter LCMV V35A, a variant virus in which the GP33 epitope is mutated was used following VV-LCMV NP priming to boost LCMV NP-specific T cells. Naïve mice infected with PR8-GP33 were used as a control in all experiments. Data are representative of 4–5 mice per group. Mice were anaesthetized using ketamine xylazine. The results are representative of three independent experiments.

We next examined whether the epitope against which the CD8+ T cells were primed impacted protection. Different epitopes have been shown to elicit varying levels of protection to the influenza virus [47]. We therefore tested whether priming with another CD8 epitope shared between X31-GP33 and PR8-GP33, the dominant D^b^-restricted NP366-374 (NP366) epitope from influenza virus nucleoprotein, could elicit better protection than the GP33 response. Mice were primed with recombinant viruses (or bacteria) expressing GP33, NP366, or a non-influenza determinant (the LCMV nucleoprotein), and challenged 30 days later. None of these approaches achieved substantial protection against PR8-GP33 rechallenge, as each group had substantial weight loss, high viral load and reduced lung function (Fig. 2A). Thus, at least for the determinants examined, the specific epitope was not a major factor in the lack of protection observed in this model system.

Next, we used a prime-boost strategy to test whether the more robust immune response induced upon boosting was superior in providing protection compared to non-boosted memory CD8+ T cells. Mice were immunized with LM-GP33 and then boosted with LCMV Armstrong 30 days after initial priming. These mice were then rechallenged with PR8-GP33 either 8 days or 30 days after the boost. We also immunized a group of mice with LCMV Armstrong and rechallenged with PR8-GP33 8 days later. The mice rechallenged with influenza virus 8 days after initial priming displayed delayed morbidity with the initiation of weight loss on ∼day 6 post rechallenge rather than at ∼day 2–3 in naïve mice (Fig. 2B). This transient delay in weight loss suggested that GP33-specific effector CD8+ T cell response present at 8 days after acute LCMV infection was capable of providing some initial protection following influenza virus infection. This group, however, still lost significant weight by day 9 post rechallenge and had reduced lung function as well as high viral load. It is worth noting that the dose of LCMV Armstrong used here has been demonstrated in our lab and others to be cleared by day 8 [48]. The prime boost group that was rechallenged 30 days after the “boost” did not experience this transient delay in weight loss and showed kinetics of morbidity similar to the LCMV Armstrong immune groups described above in terms of magnitude of weight loss and decline of lung function suggesting that the greater magnitude of GP33-specific CD8+ T cell response in this group was insufficient to mediate protection. In contrast, the prime-boost group that was rechallenged 8 days after the “boost” showed no signs of influenza-related pathology in terms of weight loss and lung function. Despite this lack of morbidity these mice still had very high viral loads until day 9 post rechallenge (Fig. 2B). While priming (and the prime-boost regimen) was able to induce a robust population of GP33-specific response capable of producing IFNγ and tumor necrosis factor (TNFα) in response to stimulation (Fig. S3), this response was still insufficient to mediate viral clearance. Thus, although this prime boost group is similar to the X31-GP33 group in terms of weight loss and lung function following rechallenge, viral control was relatively poor.

The protection from morbidity in the mice challenged 8 days after boosting was not due simply to elevated bystander inflammation as mice subjected to two other prime-boost strategies that lacked CD8+ T cells specific for influenza virus showed rapid weight loss (Fig. 2C). Furthermore, the lack of weight loss achieved by the virus-specific prime-boost strategy was recapitulated when LM-GP33 was substituted with VV-GP33 (Fig. 2C), suggesting that the lack of weight loss in these mice is not dependent on the identity of the priming agent, but on the rapid initiation of an influenza virus-specific CD8+ T cell response. These results are in line with reports that “boosted” memory CD8+ T cells are better than primary memory CD8+ T cells in controlling some acute infections [49], [50]. However, while pathology was reduced, this immune response was not sufficient to efficiently control viral load.

**Figure 3:**
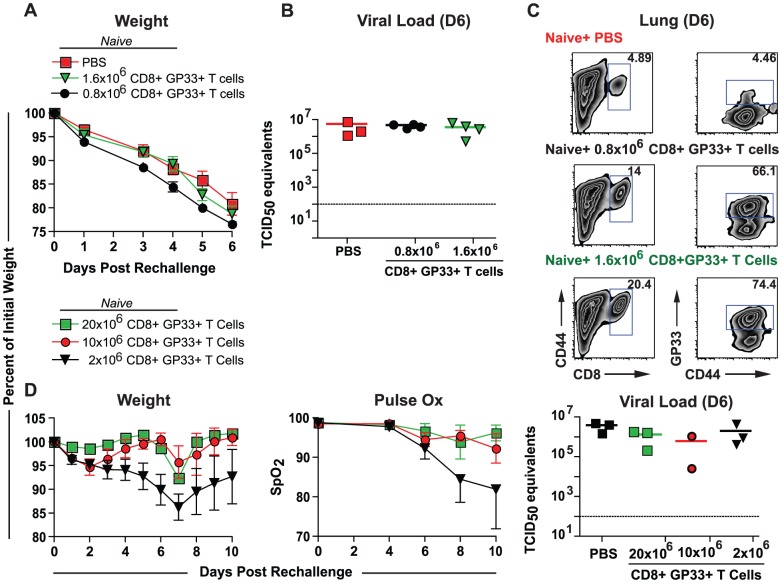
Adoptive transfer of *in vivo* or *in vitro* activated CD8+ T cells and protection from influenza virus challenge. A) Ly5.1+ mice were immunized with LM-GP33 and then boosted with LCMV Arm. On day 8 post boost the mice were sacrificed and their spleens pooled. The CD8+ T cells were then isolated using MACS columns and 1.6×10^6^ or 0.8×10^6^ GP33+ CD8+ T cells were transferred into Ly5.2+ mice. A third group was given an equal volume of PBS. All three groups were then challenged with 3 LD_50_ PR8-GP33 one day later. Weight loss after rechallenge was monitored until day 6 post rechallenge at which point the mice were sacrificed. Data are representative of at least 5 mice per group. B) Viral load in the lungs was analyzed on day 6 post challenge. C) Flow cytometric analysis of infiltrating GP33-specific CD8+ T cell in the lung was performed on day 6-post challenge. D) 20×10^6^, 10×10^6^, or 2×10^6^ *in vitro* activated GP33-specific P14 CD8+ T cells were transferred into separate groups of mice, which were then challenged with 3 LD_50_ PR8-GP33 the following day. The weight, and lung function of these mice was determined at different points following rechallenge. Viral loads were determined in the lungs at day 6 post rechallenge. Data are representative of at least 7 mice per group. Mice were anaesthetized using avertin. Results are representative of two independent experiments.

We next investigated whether we could achieve enhanced CD8+ T cell-mediated protection using adoptive transfer strategies analogous to adoptive transfer approaches used for influenza virus-specific CD4+ T cells [35], [36]. Ly5.1+ mice were immunized with LM-GP33 and 30 days later boosted with LCMV Armstrong. On day 8 following the boost, CD8+ T cells were isolated and 0.8×10^6^, or 1.6×10^6^ GP33+ CD8+ T cells were adoptively transferred to Ly5.2+ naïve mice. The recipient mice were rechallenged with PR8-GP33 the next day. Little to no protection was observed as measured by weight loss and viral load compared to a PBS-treated control group (Fig. 3A–B), despite high numbers of GP33 specific CD8+ T cells present in the lungs of these mice 6 days after rechallenge (Fig. 3C).

To determine whether the observed lack of protection was due to an insufficient number of *in vivo* primed adoptively transferred GP33-specific CD8+ T cells, we primed P14 TCR-transgenic CD8+ T cells (specific for LCMV GP33-41) using an *in vitro* approach that allowed the generation of large numbers of activated GP33-specific CD8+ T cells. We then adoptively transferred 2×10^6^, 10×10^6^ or 20×10^6^ GP33-specific CD8+ T cells into naïve mice and challenged these mice with influenza virus. Mice given 20×10^6^ or 10×10^6^ GP33-specific CD8+ T cells were almost completely protected from influenza-related morbidity and experienced virtually no decline in weight or lung function (Fig. 3D). Additionally, even the group given 2×10^6^ GP33-specific CD8+ T cells exhibited improved protection with only a 15% decline in weight and a 20% reduction in lung function. Despite the greatly reduced morbidity in these mice, the viral loads measured by qRT-PCR for viral RNA were almost indistinguishable from the PBS-treated control group. This lack of difference in viral control was confirmed using an assay for infectious virus to ensure that qRT-PCR-based approach was accurately reflecting replicating virus rather than residual viral debris or viral RNA independent of replicating virus (Fig. S4). Thus, similar to the prime-boost group described above (Fig. 2C), the immune response elicited by the adoptively transferred, *in-vitro* generated effector CD8+ T cells was insufficient to reduce viral load despite the improvement in measures of morbidity.

Overall, these results indicate that both *in vivo* and *in vitro* generated GP33-specific CD8+ T cells alone were insufficient to provide optimal protection against a pathogenic influenza virus challenge. While CD8+ T cells in large enough numbers are able to provide some protection as measured by weight loss and lung function, they are unable to significantly reduce viral load. Moreover, our results suggest that the mechanism of cross-subtype protection in X31-GP33 immune mice is unlikely to be exclusively CD8+ T cell-dependent.

### X31-GP33 mediated heterosubtypic protection is B cell dependent

**Figure 4:**
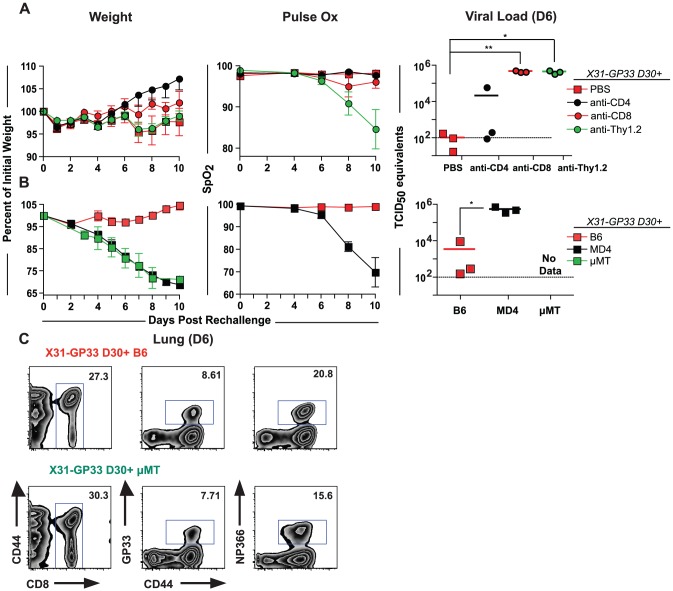
X31-GP33 mediated heterosubtypic protection is dependent on B cells. A) To determine the role of CD8+ and CD4+ T cells in X31-GP33 mediated protection, mice were primed with X31-GP33 and then rechallenged with PR8-GP33 30 days later. On days -3, -1, 1, 3, and 5 post rechallenge mice were treated with anti-CD4 (GK1.5), anti-CD8 (53.6), or anti-Thy1.2 to deplete both CD4+ and CD8+ T cells. Body weight, lung function, and viral load were determined at different time points post rechallenge. Data are representative of at least 7 mice per group. B) To elucidate the role of B cells in X31-GP33 mediated virus protection mice either lacking B cells (µMT) or mice with B cells specific for hen egg lysozyme (MD4) were used. These mice were primed with X31-GP33 and rechallenged 30 days later with 3 LD_50_ PR8-GP33. Weight loss, and lung function were examined. Viral titers were determined on day 6. Lung function and viral load were not determined for the uMT group. Data are representative of at least 4 mice per group. C) Flow cytometric analysis was performed on the lungs on day 6 to compare the GP33 and NP366-specific CD8+ T cell response in B6 and B cell transgenic mice. Mice were anaesthetized using ketamine xylazine. Results are representative of two independent experiments.

To evaluate the mechanism of cross-subtype protection in X31-GP33 primed mice, we next examined the dependence of protection in this setting on T cells (Fig. 4). CD8+ and/or CD4+ T cells were depleted from X31-GP33 immune mice prior to PR8-GP33 rechallenge with depletion being verified as >97% in the lungs. In all cases, depletion of CD8+ and/or CD4+ T cells did not increase the severity of weight loss. There was a slight but non-significant decrease in oxygen saturation following the depletion of CD8+ T cells, which was amplified when both CD4+ and CD8+ T cells were depleted (Fig. 4A). (Note, that CD4+ and CD8+ T cells were depleted simultaneously using anti-Thy1.2, which may also deplete double negative T cells, natural killer (NK) cells and innate lymphoid cells). Interestingly, the depletion of CD8+ T cells resulted in a considerable increase in viral load while CD4+ T cell depletion caused no significant difference in viral load. The viral load of the CD8+ T cell depleted group was still lower than that found in naïve mice challenged with PR8-GP33 (see Fig. 1A), although this difference was non-significant. Furthermore, the viral load was identical between the group in which only CD8+ T cells were depleted and the one in which both CD8+ and CD4+ T cells were depleted (Fig. 4A). This result agrees with previous reports that CD4+ T cells play only a minor role in modulating influenza viral titers, and that depletion of CD4+ T cells does little to alter the course of viral infection [51], [52]. While it is known that memory CD4+ T cells can cooperate with naïve B or CD8+ T cells in the context of influenza infection [36], it is unknown whether a similar cooperativity occurs with influenza virus-specific CD8+ T cells. It is interesting that CD8+ T cells in this setting were needed for control of virus while in the previous experiments (Fig. 2B, 3D) CD8+ T cells seemed to be able to control weight loss but not viral load. This difference may be due to differences in depletion versus immunization or adoptive transfer approaches or other mechanisms such as changes in immunopathology because of larger numbers of CD8 T cells suppressing other responses (e.g. CD4 T cells).

**Figure 5:**
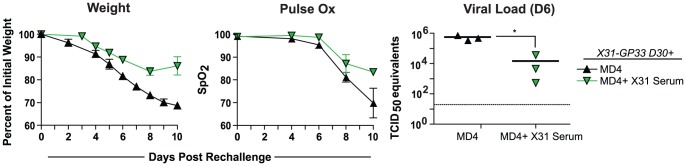
Heterosubtypic protection in B cell transgenic X31-GP33 primed mice is rescued through X31-GP33 serum transfer. To establish whether antibodies are responsible for protection in X31-GP33 primed mice, B cell transgenic (MD4) X31-GP33 immune mice were given either X31-GP33 serum or an equal volume PBS one day prior to rechallenge with PR8-GP33. Weight loss and lung function were determined at the indicated time points following rechallenge. Viral load was determined in the lungs at day 6 post rechallenge. Data are representative of at 4–8 mice per group. Mice were anaesthetized using ketamine xylazine. Results are representative of two independent experiments.

X31-GP33 immune mice are largely protected against symptoms of influenza virus infection in the absence of CD8+ and CD4+ T cells, suggesting other possible mechanisms contributing to protection. One possibility is that B cells have a role through the action of non-neutralizing antibodies specific for determinants shared between the X31 and PR8 influenza strains. To test this notion, we immunized µMT mice, which lack B cells [53], with X31-GP33. When challenged 30 days later these mice demonstrated no protection against PR8-GP33 despite a very similar influenza virus-specific CD8 T cell response compared to B6 mice (Fig. 4B and C). These data suggested that B cells are essential for heterosubtypic protection. One concern is that the immunological response may be altered in µMT mice due to the total lack of B cells. Therefore, we examined MD4 transgenic mice that have normal numbers of B cells, but have a transgenic B cell receptor specific for hen egg lysozyme [54] and are therefore unable to generate an influenza virus-specific antibody response. Similar to the µMT mice, however, MD4 mice immunized with X31-GP33 were also not protected and experienced severe weight loss upon rechallenge with PR8-GP33 (Fig. 4B). X31-GP33 immune MD4 mice also had reduced lung function and high viral load (Fig. 4B). To test whether the lack of non-neutralizing antibodies might underlie the defect in X31-GP33 immune MD4 mice, we transferred serum collected from X31-GP33 immunized B6 mice (referred to as X31 serum) into X31-GP33 primed MD4 mice and rechallenged with PR8-GP33. Protective immunity, as measured by all three parameters was improved (Fig. 5). Collectively, these data suggested that an influenza virus-specific B cell response was essential for X31-GP33 based heterosubtypic protection. As cross-neutralizing antibodies are not induced between the X31 and PR8 influenza strains [42]–[44], non-neutralizing antibodies are likely contributing to protection. One possible interpretation of the data presented thus far is that both CD8+ T cells and non-neutralizing antibodies might be necessary for optimal protection.

### Cooperativity is critical for heterosubtypic protection

**Figure 6:**
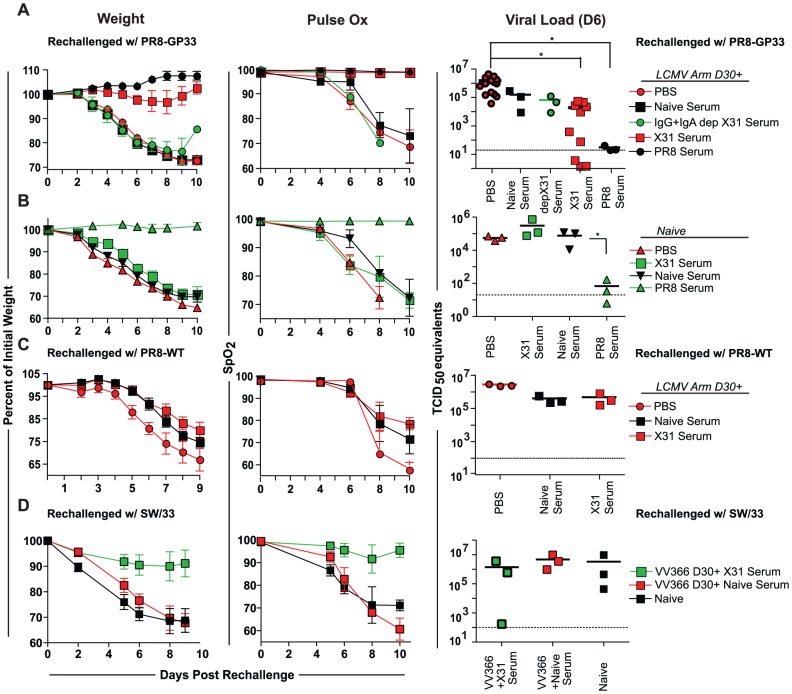
Both influenza virus-specific CD8+ T cells and antibodies are needed for optimal heterosubtypic protection. A) Mice were primed with LCMV Armstrong and then given serum from X31-GP33 immune mice, naïve mice, or PR8-GP33 immune mice 30 days later. Other groups were given X31-GP33 serum that had been depleted of IgG and IgA or an equal volume PBS. Mice were then rechallenged with 3 LD_50_ PR8-GP33 and weight loss and lung function were measured over time with the viral titer at day 6 determined. The viral load data represents the combined results from three independent experiments. B) X31-GP33, PR8-GP33, and naïve serum was also transferred into a separate group of naïve mice and weight loss and lung function were measured over time with viral titer at day 6 determined. C) LCMV Armstrong immune mice were given X31-GP33, naïve serum or PBS and challenged with PR8-WT to determine protection upon rechallenge with a virus lacking the GP33 epitope. Weight loss and lung function over time along with viral load at day 6 were determined. D) VVNP366 immune mice were given X31 or naïve serum and then challenged with SW/33 to evaluate cooperativity-based protection in non-recombinant influenza model system. Weight loss and lung function over time along with viral load at day 6 were determined. Data for all panels are representative of 6–8 mice per group. Mice were anaesthetized using avertin. Results are representative of two independent experiments.

To evaluate whether non-neutralizing antibodies in conjugation with influenza virus-specific CD8+ T cells can elicit robust heterosubtypic protection in B6 mice, we transferred serum from X31-GP33 immune mice into LCMV Armstrong immune mice. When given X31-GP33 serum, LCMV immune mice containing GP33-specific memory CD8+ T cells displayed significantly reduced weight loss and viral load compared to the LCMV Armstrong immune group that had received PBS or serum from naïve mice (Fig. 6A). LCMV immune mice that received X31-GP33 serum also maintained nearly 100% blood oxygen saturation following PR8-GP33 challenge. The protection achieved by transfer of X31-GP33 serum to LCMV Armstrong-immune mice in terms of weight loss and lung function was nearly equivalent to that achieved with transfer of serum from PR8-GP33 immune mice containing neutralizing antibodies, although PR8 serum resulted in more effective control of viral replication (Fig. 6A). To determine whether the protective factor in the serum was indeed antibodies, we administered serum that had been depleted of IgG and IgA to LCMV Armstrong immune mice and challenged with PR8-GP33 [55]. These mice exhibit no evidence of protection indicating that X31-GP33 mediated protection is antibody-dependent (Fig. 6A). While it is possible that there may be more total IgG in the transferred X31 serum compared to naïve serum, there was no obvious correlation between the total IgG levels and protection in these experiments (data not shown). Our results indicate that optimal heterosubtypic protection against influenza is elicited only when both GP33-specific CD8+ T cells and non-neutralizing antibodies are present.

While many previous studies have demonstrated that antibodies induced by X31 do not neutralize PR8 and vice versa [42]–[44], it was possible that a new neutralizing determinant might have been formed due to the insertion of the GP33 sequence into the NA stalk. Thus, we transferred X31-GP33 serum into naïve mice one day prior to challenge with PR8-GP33. These mice displayed no signs of protection and experienced severe weight loss and decline in lung function (Fig. 6B). The naïve group given PR8 serum was completely protected from challenge with PR8-GP33 due to the presence of neutralizing antibodies (Fig. 6B). This result strongly suggested that the antibodies found in X31-GP33 serum were non-neutralizing. Additionally the lack of protection found in the naïve group given X31-GP33 serum indicates that both antigen-specific CD8+ T cells and non-neutralizing antibodies were needed for protection.

To further examine the cooperativity between non-neutralizing antibodies and virus-specific CD8+ T cells in heterosubtypic protection we transferred X31-GP33 serum, naïve serum, or PBS into LCMV Armstrong immune mice. We then rechallenged these mice with PR8-WT instead of PR8-GP33. In this setting the LCMV Armstrong primed mice given X31-GP33 serum, as well as the groups given naïve serum or PBS were not protected from PR8-WT rechallenge by any measure (Fig. 6C). Thus, the protective immunity in this setting was dependent on both non-neutralizing antibodies and recognition of viral determinants by primed CD8+ T cells.

To explore whether this cooperativity-based protection could be achieved using natural influenza-virus derived epitopes we immunized mice with VVNP366 to induce an influenza virus-specific T cell response. We then waited 30 days and transferred X31 or naïve serum into these mice one day prior to challenge with the H1N1 swine influenza virus strain SW/33. SW/33 is not genetically engineered, but, like PR8-GP33, is pathogenic in mice. Similar to LCMV Armstrong immune mice, VV366 immune mice given X31 serum were protected against viral challenge in terms of both weight and lung function, with these mice also having a trend to lower viral load compared to mice given naïve serum (Fig. 6D). This finding strongly indicates that the cooperativity-based protection is not simply an artifact of out recombinant influenza virus system, but rather a likely physiologically relevant mechanism of protection against influenza virus challenge.

### Alveolar macrophages play an important role in cooperative protection

**Figure 7:**
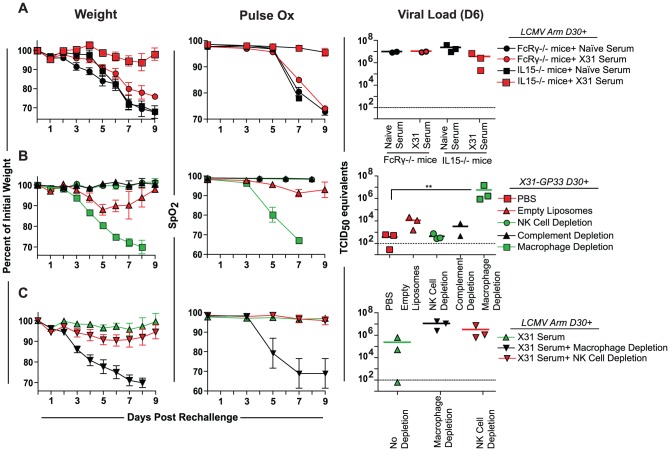
Alveolar macrophages are important for cooperative heterosubtypic protection. A) FcRγ-/- or IL-15-/- mice, which lack NK cells, were primed with LCMV Armstrong and given either naïve or X31-GP33 serum 1 day prior to rechallenge with PR8-GP33. Weight loss and lung function over time along with viral load at day 6 were determined. B) X31-GP33 immune mice were treated with clodronate i.n. to deplete alveolar macrophages, anti-NK1.1 (clone PK136) to deplete NK cells, or cobra venom factor to deplete complement. Additional mice were administered empty liposomes or PBS as controls. Mice were then rechallenged with PR8-GP33 and weight loss and lung function measured over time, and viral titer at day 6 determined. C) LCMV Armstrong immune mice were treated with clodronate liposomes or anti-NK1.1, and then given X31-GP33 serum one day prior to rechallenge. A non-treated LCMV Armstrong group was also given X31-GP33 serum. These mice were then rechallenged with PR8-GP33 and weight loss and lung function over time along with viral load at day 6 were determined. Data for all panels are representative 6–8 mice per group. Note, in some experiments where morbidity occurred, some animals where euthanized before the end of the experiment according to IACUC guidelines. Mice were anesthetized using avertin. Results are representative of two independent experiments.

The means by which non-neutralizing antibodies operate in cooperative protection is unclear. Among the possible mechanisms are: antibody-dependent cell-mediated cytotoxicity (ADCC), Fc receptor (FcR) mediated phagocytosis, and the complement pathway. To distinguish between these possibilities we used mice either lacking FcRγ or interleukin-15 (IL-15). FcRγ -/- mice are deficient in the gamma chain subunit of the FcgRI, FcgRIII and FceRI receptors resulting in functionally impaired macrophages, neutrophils, mast cells, basophils and Natural Killer (NK) cells. IL-15 -/- mice, on the other hand, are deficient in NK cells, but not these other cell types allowing the role of NK cell-mediated ADCC in heterosubtypic protection to be tested. Wild type, FcRγ -/-, and IL15 -/- mice were primed with LCMV Armstrong, rested 30 days, and then given either X31-GP33 serum or naïve serum 1 day prior to rechallenge. The LCMV immune IL-15-/- mice given X31-GP33 serum were protected upon PR8-GP33 challenge, although the decrease in viral load in these mice was only a trend. These results might reflect the moderate defect in CD8 T cell memory in these mice [56]–[58], although influenza virus-specific T cell memory in the respiratory tract appears independent of IL-15 [59]. In contrast, the FcRγ -/- mice were not protected against infection as measured by any parameters tested regardless of whether the mice were given X31-GP33 or naïve serum (Fig. 7A). Together, these results suggested that non-neutralizing antibody-based protection was FcRγ-dependent, but that NK cells were non-essential.

To further examine cooperative heterosubtypic immunity we first immunized B6 mice with X31-GP33. After 30 days we treated these mice i.n. with clodronate-loaded liposomes to deplete alveolar macrophages (AM) (and possibly other airway-resident phagocytes), cobra venom factor to deplete complement, or anti-NK 1.1 to deplete NK cells. Another group was given empty liposomes as a control. Following PR8-GP33 rechallenge, the only group left unprotected was the clodronate treated group in which AM were depleted. These mice experienced severe morbidity and high viral load despite having an unimpaired CD8+ T cell response (Fig. S5A). All other groups remained healthy and controlled the infection (Fig. 7B). This result suggested that heterosubtypic immunity mediated by non-neutralizing antibodies and CD8+ T cells was, at least in part, dependent on cells depleted by clodronate liposomes including AM. It is important to note that while we found clodronate liposome treatment to be non-toxic to uninfected mice and to result in ∼70% depletion of alveolar macrophages in the BAL fluid three days following a single clodronate treatment (Fig. S5B), it is possible that depletion of other airway populations such as dendritic cell or inflammatory macrophages could occur. The unimpaired CD8+ response seen in clodronate liposome treated mice however, suggests that any clodronate depletion of dendritic cells in the airway was insufficient to significantly impact presentation of antigen to CD8+ T cells. Furthermore, preliminary studies using adoptive transfer of alveolar macrophages obtained from the BAL of naïve mice into LCMV Armstrong immune FcRγ -/- mice suggested that reintroducing alveolar macrophages could partially rescue weight loss in half the mice when given in conjunction with X31 serum (Fig. S6). While further studies are necessary, these data are consistent with the notion that AM are involved in cooperative heterosubtypic protection.

To further evaluate the role of AM and NK cells in cooperative heterosubtypic protection, we depleted AM or NK cells in LCMV Armstrong immune mice as described above, and administered X31-GP33 serum to these mice one day prior to rechallenge. We found that only the group treated with clodronate liposomes exhibited severe weight loss and a decline in lung function (Fig. 7C). The group depleted of NK cells did display a trend towards higher viral loads then the group given only X31-GP33 serum, but this difference was not significant and this group still experienced almost no weight loss or decline in lung function, further suggesting that NK cells (or perhaps other NK1.1+ cells) only play a minor role in the mechanism of non-neutralizing antibody-based protection. Overall, this finding strongly indicates that the mechanism of non-neutralizing antibody-based protection is dependent on cells depleted by clodronate liposomes, including AM, likely through FcR-dependent AM phagocytosis or ADCC of influenza virus-infected cells.

## Discussion

The aim of universal influenza vaccination approaches is to provide long-lasting protection against a wide range of viral serotypes. Creating a universal vaccine by inducing CD8+ T cells specific for conserved internal proteins of influenza virus has received considerable attention, but remains an unrealized goal. In this study, we demonstrate that influenza virus-specific CD8+ T cells can cooperate with non-neutralizing antibodies to provide efficient cross-subtype influenza virus-specific protection. While non-neutralizing antibodies against M2e or other conserved determinants have recently been examined, our data indicate a previously unappreciated role for cooperativity between non-neutralizing antibodies and CD8+ T cell responses in the induction of optimal protection from serologically distinct influenza virus strains. This mechanism represents a novel approach by which a universal influenza vaccine could be developed.

Currently there are several promising universal influenza vaccine candidates in development. Among these candidates are broadly neutralizing antibodies, which are able to target the conserved stem region of the influenza virus [60]–[64]. These broadly neutralizing antibodies have been found to be cross reactive among different H1 or H3 influenza subtypes and are likely to represent a major advance in generating more effective influenza virus vaccines. However, current antibodies specific for the H1 stem are largely effective only against heterologous H1 and H5 viruses, and antibodies against the H3 stem are only effective against H3 viruses [60]. Interestingly, neutralizing antibodies were sometimes induced following vaccination with a pandemic H1N1 vaccine, but were of too low magnitude to induce robust heterosubtypic protection [64]. Until neutralizing antibodies can be generated against an antigen conserved between many different influenza subtypes, humans will remain vulnerable to the threat of a pandemic from a novel influenza strain such as H7N7, H9N2, etc. [65]. Another promising potential universal influenza vaccine targets the ectodomain of matrix protein 2 (M2e) [66]. The M2e sequence is conserved across influenza virus subtypes, and humoral anti-M2e immunity has been shown to protect against influenza virus challenge in mice [67], [68]. However, M2e-based protection does not prevent or resolve infection and is of a lower potency that HA-specific antibodies, making an M2e-dependent therapy more likely to act as a safety net in the case of the emergence of pandemic influenza strains rather than a replacement for current vaccines [69]. One concern associated with both broadly neutralizing antibodies and M2e based vaccines is that widespread use of these vaccines will introduce immune pressure promoting the evolution of antigenic escape viruses [66]. There have already been reports of escape viruses being generated in response to M2e antibodies, with one study finding that virus mutants with antigenic changes in M2e emerged in 65% of virus-infected mice treated with anti-M2e, although some level of protection remained despite these mutations [69]. An interesting avenue of future research will be to determine if cooperativity between T cells and non-neutralizing antibody can be used to boost the protection elicited through these vaccination strategies. Virus-specific CD8+ T cells do not seem to be generated by HA-stalk or M2e immunization strategies [70], so a vaccine in which CD8+ T cells can be elicited specific for conserved influenza virus determinants, combined with approaches to generate HA-stalk or M2e targeting antibodies, may offer improved protection. Furthermore, the overlapping protection provided by virus-specific CD8+ T cells should help reduce the possibility of an escape virus emerging.

Recent reports have implicated NP-specific IgG in heterosubtypic immunity to influenza virus [28], [29]. This previous work found that NP protein was detectable in the BAL and nasal washes of influenza virus-infected mice, thereby allowing the NP antigen to interact with NP-specific antibodies and form complexes to stimulate antiviral immune responses. These studies also demonstrated that 5 daily antibody injections starting 3 days prior to infection were required to reduce viral load in naïve mice, using a 0.25 lethal dose 50% (LD_50_) influenza rechallenge. Our findings extend this work in determining that cooperativity between influenza virus-specific CD8+ T cells and antibodies is important for heterosubtypic protection in immune competent mice. NP-specific antibodies are likely to be a primary component of the X31-GP33 serum used in our work. The cooperativity we demonstrated may mean that excessively high amounts of non-neutralizing antibodies might not be required if virus-specific CD8+ T cells are also present. Since previous influenza virus infections or vaccinations have likely induced anti-NP antibodies and influenza virus-specific memory CD8+ T cells in most adults, it is interesting that more heterologous protection does not seem to exist in humans. One reason may be that sufficiently high titers of anti-NP antibodies are not present in adults to mediate cooperative protection. Indeed one report indicates that trivalent inactivated influenza virus vaccine (TIV) only rarely and modestly boosted existing levels of anti-NP IgG [32]. Alternatively, perhaps such cooperative immunity is, in fact, one reason for the relatively low mortality in healthy adults for most strains of influenza virus. Our results suggest that it will be interesting to test whether this cooperative immunity might wane with increasing age, a theory supported by several reports [26]–[29]. It is possible that the lack of CD8 T cell boosting by the yearly vaccine allows CD8 T cell memory to decline over time even in healthy young adults. Future studies will be necessary to test some of these ideas in humans.

The mechanism by which non-neutralizing antibodies operate in the setting of heterosubtypic immunity remains poorly understood. Unlike neutralizing antibodies, non-neutralizing antibodies do not prevent viral entry into host cells and must therefore employ a different means of action to reduce viral load. FcRs have been reported to be important mediators of this process, but the specific cell types directly involved in reducing influenza viral load and pathology by this mechanism are unclear. Alveolar macrophages have been shown to play a critical role in influenza virus protection, likely through ADCC or phagocytosis [71], [72]. Some reports have also suggested that NK-cells may be involved in influenza virus protection through ADCC [59], although several recent studies have found this to be unlikely [73], [74]. The complement pathway could also have an important role due to the ability of complement to bind and lyse infected cells or enveloped virus in the presence of antibodies. Complement has been demonstrated to be able to neutralize the influenza virus in the presence of natural antibodies [75], and complement component C3 may be important in T cell priming and migration to the lungs [76]. Indeed, C3 deficiency in humans correlates with recurrent infections of the upper and lower respiratory tract [77]. In the current studies we found that heterosubtypic protection was dependent, at least in part, on alveolar macrophages. Intranasal administration of clodronate liposomes have been shown to selectively deplete alveolar macrophages while leaving the interstitial macrophage population as well as other cell types in the lungs intact [71], [78]–[80]. However, the possibility of off target effects of the clodronate liposome approach cannot be fully excluded. The unimpaired CD8+ T cell response found in clodronate-treated mice (Fig. S3A) however, suggested that depletion of dendritic cells was unlikely to be responsible for the lack of protection in this setting. Thus, alveolar macrophages are likely a major cell type impacted by this treatment and are expected to act to help reduce viral load through recognition of the Fc region of non-neutralizing antibodies. This recognition can lead to ADCC and/or antibody-dependent cell-mediated phagocytosis directed against infected cells bound by non-neutralizing antibodies.

The reduced effectiveness of seasonal influenza vaccines and greater infection-related morbidity and mortality in the elderly is thought to be due to alterations in both the innate and adaptive immune response that occur with age [3], [81]–[84]. Among the alterations reported in the elderly that could influence immunity to infection are changes in macrophages, NK cells, neutrophils, pathogen recognition via Toll-like receptors, innate cell cytokine production [85], [86], as well as decreased numbers, proliferation and signaling of B and T cells [87]–[91]. Interestingly, there have also been reports of changes in FcRs that occur with age, which could lead to defects in FcR-dependent effector functions [92]. While it has been shown that CD8+ T cell and neutralizing antibody-based protection obtained at a young age is still protective many years later [93]–[95], less is known about non-neutralizing antibody-dependent protection. Since this type of protection relies on FcR-dependent effector mechanisms to clear infected cells it will be important to determine if the heterosubtypic protection observed in young mice is also seen in aged groups of animals. A major goal of “universal” influenza vaccines is to elicit cross-subtype influenza virus protection in both young and aged populations. Hence, age-related defects in the immune system are a critical issue that must be addressed in future studies to determine if cooperative protection is an effective strategy in eliciting heterosubtypic influenza protection.

Collectively, we have shown that influenza virus-specific CD8+ T cells in cooperation with non-neutralizing antibodies are able to provide optimal protection against a lethal influenza virus rechallenge. This protection is only exhibited when both influenza virus-specific CD8+ T cells and non-neutralizing antibodies are present. Furthermore, non-neutralizing antibodies likely contribute to influenza virus clearance, possibly through a mechanism involving alveolar macrophages. It should be pointed out that while we have focused largely on CD8+ T cells, it is possible that cooperative protection will occur for CD4+ T cells and non-neutralizing antibodies. Indeed, there is good evidence that CD4+ T cells can contribute to protective immunity to influenza virus [35], [96], [97] and cooperate with naïve B and CD8+ T cells [36]. It will be important to address this issue in the future. This work provides novel insights into cross-subtype influenza virus protection and could have implications for the development of a universal influenza vaccine.

## Materials and Methods

### Ethics statement

This study was carried out in accordance with the recommendations in the Guide for the Care and Use of Laboratory Animals of the National Institutes of Health. Protocols were approved by the Institutional Animal Care and Use (IACUC) committees of the Wistar Institute, (animal welfare assurance number A3432-01) or University of Pennsylvania (animal welfare assurance number A3079-01). The Wistar and University of Pennsylvania Animal Care and Use Programs are fully accredited by the Association for Assessment and Accreditation of Laboratory Animal Care International (AAALAC).

### Mice and viruses

C57BL/6 and Ly5.1+ mice were purchased from the National Cancer Institute (Frederick, MD) or Jackson Laboratories (Bar Harbor, ME). Age- and sex matched IL-15-/- mice were obtained from Taconic (Germantown, NY), FcRγ knockout mice (FcRγ KO; strain name B6.129P2-*Fcer1g^tm1Rav^*N12) were purchased from Taconic Farms, Inc. (Hudson, NY), and B cell-deficient B6.129S2-*Igh^tmICgn^*/J (µMT) mice and anti-HEL B-cell receptor (BCR)-transgenic C57BL/6-TgN (IghelMD4) mice (referred to as MD4) were obtained from the Jackson Laboratory. For primary or secondary infections, mice were inoculated using the following pathogens, doses and routes: with LCMV Armstrong (LCMV Arm; 2×10^5^ PFU i.p. or 5×10^4^ PFU i.n); recombinant X31 influenza virus expressing the LCMV GP33 epitope (X31-GP33; 1.6×10^5^ TCID_50_ i.n.); vaccinia virus (VV) expressing the LCMV GP33 epitope (VVGP33), VV expressing the influenza virus NP366 epitope (VVNP366), and VV expressing LCMV Nucleoprotein (VV-NP (LCMV)) all used at 3×10^5^ PFU i.n.; Listeria (LM) expressing the GP33 epitope (LM-GP33; i.v.); Vesicular stomatitis virus expressing the ovalbumin (OVA) epitope (VSV-OVA; 2×10^6^ PFU i.v.); LCMV Armstrong V35A which lacks the GP33 epitope (LCMV-V35A; 2×10^5^ PFU i.p.). For rechallenge experiments, mice were given either recombinant PR8 influenza virus expressing the LCMV GP33 epitope (PR8-GP33; 3 LD_50_ i.n.); wild type PR8 influenza virus (PR8-WT; 3 LD_50_ i.n.); wild type swine influenza virus (SW/33; 3 LD_50_ i.n). For both strains of PR8 1LD_50_ = ∼250 TCID_50_. Prior to i.n. infections, mice were anesthetized by i.p. injection of ketamine hydrochloride and xylazine (Phoenix Scientific, San Marcos, CA) in 0.2 ml Life Technologies HBSS (Invitrogen, Carlsbad, CA). In some experiments, mice were anesthetized with 2.5% Avertin (0.2–0.35 ml) i.p. Recombinant influenza virus strains containing the LCMV GP33–41 epitope inserted in the neuraminidase stalk region were obtained from Dr. Richard J. Webby (St. Jude Children's Research Hospital, Memphis, TN) and have been previously described [35], [36]. These viruses were propagated in eggs, and stored at −80°C. The replication and pathogenicity of these recombinant X31 and PR8 strains were not substantially different from their nonrecombinant counterparts (data not shown). Viral titers were determined by plaque assay on Vero cell monolayers (for LCMV and VV) or on Madin-Darby canine kidney cell monolayers (for X31-GP33 and PR8- GP33) as previously described [98]. For all experiments, naïve C57BL/6 mice were also infected with influenza virus to allow comparison of weight loss, viral load, and pulse oximetry between different experiments. The concentration of infectious virus in lungs was determined by titration of homogenized tissues in Madin-Darby canine kidney cell (MDCK) microcultures as described previously [6]. Lung titers are expressed as dilution of lung extract at which 50% of the MDCK cultures revealed virus growth (TCID_50_/ml).

### Adoptive transfers

For all adoptive transfer experiments, congenic mice differing in Ly5 (Ly5.1 versus Ly5.2) were used. For adoptive transfers CD8+ T cells were purified (90% purity) using magnetic beads (CD8+ T cell isolation kit, MACS beads; Miltenyi Biotec, Auburn, CA).

### Measurement of pulse oximetry

The MouseOx Pulse-oximeter (Starr Life Sciences, Oakmont PA) was used to measure blood oxygen saturation (SpO_2_) in PR8-GP33 infected mice during the course of infection. A depilatory agent (Nair, Church & Dwight Co.) was applied to the neck of anesthetized mice 1 day prior to influenza infection to remove hair and delay future hair growth. For readings, the oximeter clip was placed on the neck and percent SpO_2_ was measured each second over several minutes, data shown is the average of SpO_2_ readings recorded over 3–5 minutes per mouse.

### Adoptive transfer of alveolar macrophages

Alveolar macrophages were isolated and transferred as previously described [71]. Briefly AM were isolated from BAL with PBS-EDTA from C57BL/6 mice. 10 mice were sacrificed as donors for every recipient mouse. A 23-gauge cannula was inserted into the trachea, and cells were collected by washing the airway lumen with 3×0.5 ml PBS-EDTA. The obtained BAL fluid was centrifuged, and cells were washed twice with PBS, counted, and resuspended in PBS. 4.5×10^5^ cells were then transferred i.n. into recipient mice at a volume of 50 ul/mouse.

### *In vivo* depletion of immune cells

NK1.1 cells were depleted *in vivo* by i.p. injection (0.2 mg/injection) of rat mAb PK136. CD8+ T cells were depleted by i.p. injection of rat mAb 53.6, CD4+ T cells were depleted using rat mAb GK1.5 and both CD4+ and CD8+ T cells were depleted simultaneously using anti-Thy1.2 (0.2 mg/injection; clone 30H12, isotype Rat IgG2b obtained from BioXCell). All antibody treatments were givens days -3, -1, 2, and 5 post PR8-GP33 rechallenge. Depletion was confirmed by flow cytometric analysis on day 6 post rechallenge in the lungs. All *in vivo* mouse antibodies were purchased from Bio X Cell (West Lebanon, NH).

### Depletion of alveolar macrophages *in vivo*

Alveolar macrophages were depleted using the liposome-mediated macrophage depletion technique based on the intracellular delivery of the drug dichloromethylene diphosphonate (clodronate). Preparation of clodronate-liposomes and applications of the technique was done as previously described [79]. Alveolar macrophages were depleted by i.n. administration of 50 ul of clodronate-liposomes on days -3, -1, and 2 post PR8-GP33 rechallenge.

### RNA isolation and real-time quantitative PCR

Viral quantitative real-time RT-PCR was performed essentially as previously described [26]. Briefly, total RNA was purified from lungs of PR8-GP33 infected mice using the RNeasy Mini Kit (Qiagen, Valencia, CA). Reverse transcriptions were primed with random primers and performed using the High Capacity cDNA Reverse Transcription Kit from Applied Biosystems (Foster City, CA). Real-time quantitative PCR (qRT-PCR) was performed on cDNA using TaqMan Universal PCR Master Mix (Applied Biosystems) and probes and primers specific to the influenza PA protein with all samples analyzed in triplicate. Reactions were run on a real-time PCR system (ABI7500; Applied Biosystems). Amount of influenza viral RNA per sample was then calculated using known standards. The total amount of virus per lung was then calculated using the mass of the lung portion taken for viral RNA determination in relation to the total lung mass. The TCID_50_ of each sample was determined by calculating the volume of virus per lung (using the viral RNA determination of the PR8-GP33 stock) and then calculating the total TCID_50_ in the lungs using the known TCID_50_ per unit volume of the viral stock. The limit of detection was determined by performing qRT-PCR on lung samples from uninfected mice and represented by a dashed line.

PA sense: CGGTCCAAATTCCTGCTGAT. PA antisense: CATTGGGTTCCTTCCATACA. PA probe: 6FAMCCAAGTCATGAAGGAGAGGGAATACCGCTTAMRA

### Isolation of lymphocytes from tissues

Lymphocytes were isolated from tissues as previously described [99]. Briefly, mice were euthanized and the hepatic vein cut. Lungs were perfused by injection of PBS into the hepatic artery or the right heart ventricle. Lungs were cut into pieces and incubated in 0.2 mg/ml collagenase D (Roche Diagnostic, Indianapolis, IN) at 37°C for 35 min. Spleens and lymph nodes were homogenized using a cell strainer. In all tissues, red blood cells (RBCs) were lysed using ACK lysing buffer (Quality Biologicals, Gaithersburg, MD), and lymphocytes were washed and counted.

### Serum transfers

Serum was collected from naïve and day 30+ X31-GP33, or PR8-GP33 infected mice. Serum samples from individual mice were pooled and 1 ml of pooled serum/mouse was injected i.p. into mice on day -1 prior to PR8-GP33 rechallenge. In some instances to verify that the antibodies present in the serum were responsible for any protective effects, serum was depleted of IgG and IgA using Protein A and G SpinTrap (GE Healthcare, Pittsburgh, PA) according to manufacturer's instructions.

### *In vitro* stimulation of antigen-specific T cells

Effector CD8 T cells were generated *in vitro* by peptide-stimulation of TCR-transgenic splenocytes (obtained from a P14 transgenic mouse) specific for the LCMV glycoprotein peptide (P14 mice specific for GP33-41). Briefly, spleen cells were incubated with 5 µM GP33 peptide for two hours. The peptide was washed off, media replaced and the cells were cultured for 48 hrs in 24-well plate, and maintained afterwards in 75T culture flasks in IL-2 - supplemented media for 5 days. The media was changed every 48 hours. A daily sample from the culture was examined by flow cytometry for the expression level of the activation markers, CD44 and CD25. On day 5, the cells were harvested, washed in PBS, counted and resuspended in PBS for adoptive transfer.

### Flow cytometry intracellular cytokine staining

Lymphocytes were stained using standard techniques and analyzed by flow cytometry. Virus-specific CD8 T cells were quantified using MHC class I peptide tetramer staining. MHC class I peptide tetramers were made and used as described [98]. Antibodies to CD8 and CD44 were purchased from eBioscience (San Diego, CA). Staining and analysis were performed as previously described [92]. Function was investigated by intracellular cytokine staining following antigen stimulation (IFNγ, TNFα, IL-2, CD40L). Briefly, 1×10^6^ splenocytes were cultured in the absence or presence of the indicated peptide (0.2 mg/ml) and brefeldin A for 5 h at 37°C. Influenza virus pooled peptides were used to evaluate the influenza virus-specific CD8+ T cell responses. This pool contains 147 overlapping peptides from influenza virus NP and M proteins, and we also included the GP33 peptide in this pool. For later experiments the overall influenza-specific CD8+ T cell response was evaluated via intracellular cytokine staining following stimulation with peptides from the influenza proteins HA, NA, NS1, NS2, PA, PB, NP, as well as the LCMV epitope GP33. Following staining for surface antigens as described above, cells were stained for intracellular cytokines using the Cytofix/Cytoperm kit (BD Biosciences). Samples were collected using an LSRII flow cytometer (BD Biosciences).

### Statistical analysis

Results represent the mean ± SEM unless indicated otherwise. Statistical significance was determined by paired or unpaired Student's *t* test. Statistical analyses were performed using Prism GraphPad software v5.0. (*, p<0.05; **, p<0.01; ***, p<0.001).

### Accession numbers

Neuraminidase-956530; Interferon gamma-15978; Tumor necrosis factor-21926; Hemagglutinin- 956529; Nucleoprotein (Influenza)-956531; Nucleoprotein (LCMV)-956592; Non-structural protein 1–956533; Non-structural protein 1–956532; Polymerase acidic-956535; Fc receptor-109615; Glycoprotein (LCMV)-956590; Matrix protein 2–956528; Interleukin 15–16168. All accession ID numbers are recorded from the Entrez Gene database.

## Supporting Information

Figure S1 **Protection against influenza virus rechallenge is independent of the magnitude of virus-specific CD8+ T cell response.** A) C57BL/6 mice were primed with either X31-GP33 i.n. or LCMV Armstrong i.p. These mice, as well as a naïve control group, were rechallenged on day 30+ following primary infection with 3 LD_50_ influenza PR8-GP33 virus. The weight loss after rechallenge and lung function (as measured by pulse oximetry) was assessed. Mice were sacrificed on day 0, 4, 6, and 9 post rechallenge and viral titers were determined in the lungs. Data are representative of 9 mice per group with three mice sacrificed at each time point for viral load determination. B) The spleen and BAL were analyzed by flow cytometry. Intracellular staining (ICS) using a pool of overlapping NP and M peptides plus GP33 was carried out to determine the magnitude of the influenza virus-specific CD8+ T cell response in various tissues with cells that were IFNγ+ defined as influenza-virus specific. C) Surface staining was also performed to verify the frequency of the GP33-specific CD8 response in the lungs. Mice were anaesthetized using avertin.
(EPS)

Figure S2 **Improved viral control following influenza virus infection after intranasal priming.** C57BL/6 mice were primed with LCMV Armstrong i.n. or i.p. 30 days later these mice along with a group of naïve controls were rechallenged with PR8-GP33. Weight loss, lung function, and viral load at the indicated time points post infection was then determined. Data are representative of 9 mice per group with three mice sacrificed at each time point for viral load determination. Mice were anaesthetized using ketamine xylazine. Results are representative of three independent experiments.
(EPS)

Figure S3 **Magnitude of the GP33-specific CD8+ T cell response following different immunization regimens.** C57BL/6 mice were primed with LCMV Armstrong i.n. and rechallenged either 8 or 30 days after primary infection. Other groups were infected with LMGP33 and boosted with LCMV Armstrong i.n. at memory time point. These mice were then rechallenged with PR8-GP33 8 or 30 days after boosting. Mice in each group were sacrificed on D0, 4, 6, and 9 following rechallenge and intracellular staining performed on cells from the lungs. Responses to the GP33 epitope were used to establish the kinetics of the GP33-specific immune response and the ability of these cells to produce the cytokines IFNγand TNFα. Mice were anaesthetized using ketamine xylazine. Data are representative of three independent experiments.
(EPS)

Figure S4 **Viral load as determined by infectious virus assay matches viral load determined by qRT-PCR.** 20×10^6^, 10×10^6^, or 2×10^6^ *in vitro* activated GP33-specific P14 CD8+ T cells were transferred into separate groups of mice, which were then challenged with 3 LD_50_ PR8-GP33 the following day. Viral loads were determined in the lungs at day 6 post rechallenge. Both an assay of infectious virus and RT-PCR were used to determine viral load. Mice were anaesthetized using avertin. Results are representative of two independent experiments.
(EPS)

Figure S5 **Clodronate treatment results in depletion of AM and an unimpaired CD8+ T cell response.** A) C57BL/6 mice were primed with X31-GP33 i.n. and rechallenged 30 days later with PR8-GP33. Mice were treated on D-3, -1, and 2 with clodronate or plain liposomes delivered intransally. Mice were then sacrificed on day 6 post infection and the lung CD8+ T cell response was examined by flow cytometric analysis. Tetramer staining was used to assess the GP33-specific response in these mice. B) Naïve mice were treated with clodronate or plain liposomes. Three days later the mice were sacrificed and the BAL wash was taken to evaluate AM depletion in these mice. The percent AM depletion was determined relative to the AM level in naïve PBS treated mice. AM were defined as CD3-CD19-CD11b-MHCII-CD11c+. Mice were anaesthetized using avertin. Results are representative of two independent experiments.
(EPS)

Figure S6 **Alveolar macrophage transfer seems to partially rescue influenza infection induced weight loss.** A) FcRγ-/- mice were immunized with LCMV Armstrong i.n. 30 days later these mice were given X31, or naïve serum. Additionally, some groups of mice were given alveolar macrophages obtained from naïve mice. The next day all groups were infected with PR8-GP33 and weight loss following rechallenge was assessed. Mice were anaesthetized using avertin.
(EPS)
